# Polyphosphate Kinase from *M. tuberculosis*: An Interconnect between the Genetic and Biochemical Role

**DOI:** 10.1371/journal.pone.0014336

**Published:** 2010-12-15

**Authors:** Vijayalakshmi Jagannathan, Parvinder Kaur, Santanu Datta

**Affiliations:** AstraZeneca India Pvt. Ltd., Bangalore, India; University of California Merced, United States of America

## Abstract

The enzyme Polyphosphate Kinase (PPK) catalyses the reversible transfer of the terminal γ-Pi of ATP to form a long chain Polyphosphate (PolyP). Using an IPTG inducible mycobacterial vector, the vulnerability of this gene has been evaluated by antisense knockdown experiments in *M. tuberculosis*. Expression profiling studies point to the fact that down regulation of PPK caused cidality during the late phase in contrast to its bacteriostatic mode immediately following antisense expression. PPK thus seems to be a suitable anti-tubercular drug target. The enzyme which is a tetramer has been cloned in *E. coli* and purified to homogeneity. An enzyme assay suitable for High Throughput Screening was optimized by using the statistical Taguchi protocol and the kinetic parameters determined. The enzyme displayed a strong product inhibition by ADP. In order to accurately estimate the product inhibition, progress curve analysis of the enzyme reaction was monitored. The kinetic equation describing the progress curve was suitably modified by taking into account the product inhibition. The reversible nature of the enzyme indicated a possibility of a two way ATP↔ADP switch operating in the bacteria as a response to its growth requirement.

## Introduction

Inorganic Polyphosphate (PolyP), a likely precursor in pre-biotic evolution, is a ubiquitous entity that is found in diverse locations such as volcanic condensates, deep oceanic steam vents and inside living cells. They are linear polymers in the size range of 3–1000 of orthophosphate residues linked by high-energy phospho-anhydride bonds. Though this polymer was identified more than a century ago, its biochemical role has only been clarified in the last decade through the pioneering work by Kornberg and colleagues [1]–[4].

In nature, polyphosphate is formed by dehydration of orthophosphate at elevated temperature, while its cellular synthesis is catalyzed by the enzyme Polyphosphate Kinase (PPK) which uses the γ-Pi of ATP to extend the polymer. Its reverse reaction is the formation of ATP from ADP and Pi [2]. Although this polymer is found in nearly all types of bacteria, its level fluctuates orders of magnitude depending on the physiologic and metabolic state of the cell. Its cellular accumulation is in response to specific physiological states - like deficiencies in amino acid, Pi, nitrogen or to the more general stresses akin to a nutrient downshift or high salt. It seems that the intracellular polyphosphate level is maintained by shifting the equilibrium between the forward and the reverse rate of the enzyme reaction. An interesting observation has been the stage specific essentiality of the gene. Mutants of *E. coli* (*ppk* ^−^) have severe growth defects in the stationary phase while showing normal growth kinetics in the logarithmic zone. Stationary phase of growth as studied in the laboratory can be thought of resembling the stressful and deprived state that characterizes the natural habitat of most bacteria. To cope with such adverse conditions, stationary phase cells undergo drastic physiological and morphological changes and a number of genes are induced in this phase in order that the cells survive [5].

Localization of PolyP granules in the vicinity of bacterial nucleoid suggests their possible involvement in regulation of gene activity, which may be essential for adaptation to stationary phase and other stresses [6]. Thus, not only polyphosphate could act as an essential marker for stress response, but it also might supply the organism of the much needed ATP to cope during its nutrient downshift or environmental stress. Additionally, the phosphoanhydride bonds can be used as a high energy source for phosphorylation of glucose as its free energy of hydrolysis is almost equal to that of ATP. PPK has also been implicated in virulence and *in-vivo* growth in several bacteria. In view of the phylogenetic similarity of the enteropathogens and the frequency with which virulence factors are expressed in stationary phase, mutants lacking the *ppk* gene in pathogenic *Shigella flexneri*, *Salmonella enterica serovar dublin*, and *Salmonella enterica serovar typhimurium* showed decreased virulence phenotype such as: (*i*) growth defects, (*ii*) defective responses to stress and starvation, (*iii*) loss of viability, (*iv*) polymyxin sensitivity, (*v*) intolerance to acid and heat, and (*vi*) diminished invasiveness in epithelial cells. A *ppk* mutant of *Pseudomonas aeruginosa* was shown to be defective in quorum sensing and the dependent virulence factors, elastase and rhamnolipid; the mutant was also deficient in biofilm formation and was not lethal in a burned-mouse pathogenesis model [7]. *Vibrio cholerae ppk* mutants show defects in growth, motility, and surface attachment, features linked to virulence [8], [9].

Of all the pathogenic bacteria *M. tuberculosis* stands out as the most successful example of survivability under *in-vivo* and *in-vitro* stress. In a landmark experiment by Corper and Cohn in 1920, it was shown that sealed cultures of TB bacilli at 37°C was viable (ranging from 0.1% to 1%) even after 12 years [8]. It is of interest to note that the bacilli are adept at withstanding insults such as desiccation, nutrient deprivation and osmotic shock. Also the in-vitro stationary state grown bacilli and the one directly derived from mouse lung are able to survive heat exposure at 53°C much better that the logarithmic phase grown bacteria [10].

The persistent tubercular bacilli in the human host are often impervious to standard anti-TB drugs which are generally focused on the rapidly dividing phase of the organism. Thus making the treatment of TB, in its short term version of six months, the longest among all anti-infectives. We postulate that PPK in *M. tuberculosis* could be one of the key enzymes that harness the bacteria to survive the onslaught of stress and nutritional downshift and persist in the in-vivo milieu evading the immune system. Potent inhibitors of M.tu PPK when combined with the standard anti-TB regimen may thus be able to substantially reduce the duration of therapy. An anti-mycobacterial drug targeted against PPK should also show selectivity and less toxicity, as the enzyme has not been found in mammalian cells. To test this hypothesis conditional induction/knockdown strategies can be used to simulate altered physiological states and one could measure the resulting effects, be metabolic changes or survival characteristics. A crucial index of a target gene is whether its inhibition leads to stasis or cidality. We have recently reported the down regulation of various anti-tubercular target genes using inducible anstisense RNA technology [11]. It would be interesting, given the biochemical understanding of PPK, to investigate into the survival kinetics of *M. tuberculosis* following knockdown of *ppk*.

The aim of the present study was to observe the effect of modulation of *ppk* and evaluate its viability as a drug target. If viable, characterize the enzyme from the perspective of its biological role and inhibitor screening.

## Results

### Vulnerability of PPK in *M. tuberculosis*

**Figure 1:**
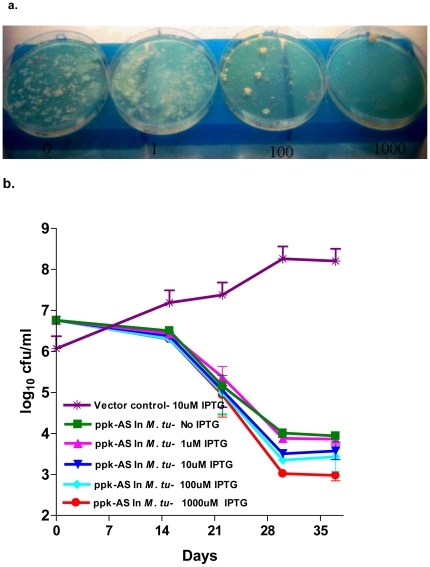
Knockdown of PPK in *M. tuberculosis*. (**a**) *ppk* antisense under IPTG inducible promoter was transformed. The transformants were divided equally and plated under various concentration of IPTG (0–1000 uM) (**b**) ppk antisense were induced in cultures at various IPTG concentration (0–1000 uM) in a liquid culture. It was plated for cfu enumeration of the survivors after various time intervals.

Antisense expression of *ppk* clearly shows that the gene is essential in *M. tubercuolsis* (Figure 1). Whereas the antisense transformants in the plates show confluence in absence of the inducer IPTG, there was only a single colony at the maximum inducer concentration of 1 mM. The intermediate concentration of IPTG show graded number of transformants indicating a dose response (Figure 1a). Though it is difficult to quantify the level of knockdown of gene expression following antisense expression, the qualitatively proportional relationship between IPTG concentration and cfu indicated, that so far as the growth of the bacteria in an agar media is concerned, the gene was vulnerable. This indicated that a moderate down regulation of the gene expression inhibited the growth. The effect of antisense expression in the liquid media showed somewhat different response (Figure 1b). Till 15 days following the antsisense expression there was only a marginal drop in cfu. However in the next 15 days there was 3–4 log reduction in the cfu indicating a steep bactericidal dose response. This drop compared favorably with our earlier observation of 2–3 log drop in cfu with proven antitubercular targets like rpoB, gyrA/B and inhA [11]. That these effects were not compounded by the long term plasmid stability were verified in an independent experiment. It was seen that the plasmid pAZI9018b was stable in mycobacteria even in the absence of antibiotic pressure for over two months (data not shown).

### Fold changes in the expression of *M. tuberculosis ppk* during different phases of growth

The expression profile of *ppk* indicated that compared to the constitutive gene *SigA*, *ppk* is over expressed in the log phase and there is approximately a ten fold down regulation in the stationary phase (Table 1). This is in alignment with the level of ATP during these phases. We speculate that the forward reaction is predominantly operative during the log phase while the reverse is operative during the stationary phase. A recent data indicate that the FoF1 ATPase inhibitor TMC 207 was inactive in the stationary phase though it had less (five fold) ATP [12]. This data apparently looks counterintuitive, however in light of our hypothesis that ATP is generated, primarily through reverse PPK activity from polyphosphate and ADP in stationary bacteria, it seems logical for TMC207 to loose its potency in the stationary phase. We observed that the level of *ppk* transcript in the stationary phase antisense strain was twice the level of wild type strain (Table 1). It should be noted that we had done a single tube RT-PCR where both the primers (forward and reverse) are present. This would imply that both sense and antisense transcript would be equally amplified. The fact that the concentration of the *ppk* transcript in the stationary phase antisense strain was twice the level of wild type strain would indicate that in the former both the sense and the antisense are in equal amount while in the wild type only the sense mRNA was transcribed. That the concentration of sense and antisense transcripts were equal would indirectly indicate that they form a 1∶1 hybrid. Additionally, we could not see any amplification in the wild type RTPCR when using the forward primer in the reverse transcriptase assay (data not shown). This indicated that the antisense transcript is only produced in the strain harboring the antisense construct.

**Table 1:** Fold changes in the expression of *M. tu ppk* in different growth phases.

	Log phase(5 days)	Stationary phase(28 days)	Stationary phase(63 days)	Antisense(63 days)
***Sig A***	1.01±0.17	0.99±0.08	1.00±0.14	1.01±0.23
***ppk***	28.8±2.3	2.45±0.28	2.9±0.15	5.35±1.08

### Cloning, expression and Purification of PPK

**Figure 2:**
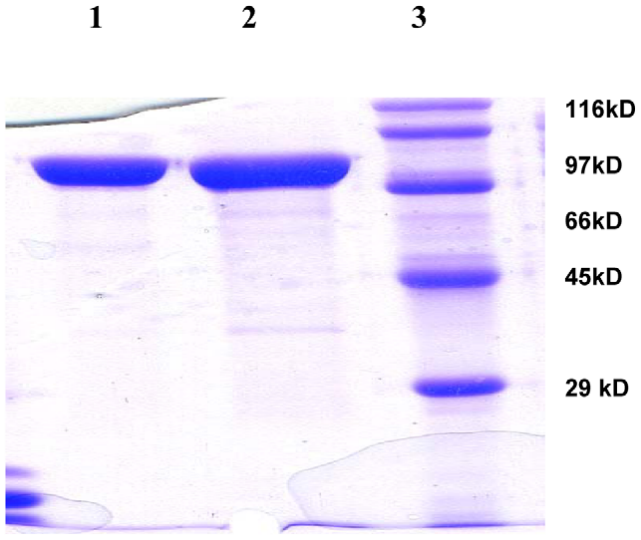
SDS-PAGE analysis of purified PPK. Samples of PPK after purification were analyzed by SDS-PAGE (10%) and visualized by Coomassie blue staining; lane 1- PPK His-tagged (purified using Ni-NTA), lane 2- PPK untagged (purified using Mono-Q), lane 3- MW marker (Biorad-6H).

The standard 37°C expression of PPK with clone pAZ1 or pAZ2 generated largely inclusion bodies. However soluble expression was achieved by changing the growth temperature from 37°C to 20°C. Induction with IPTG was also not required. Overnight growth at 20°C enhanced expression of PPK in the soluble form. Fortunately, the initial step of Ammonium sulphate fractionation (35% v/v) resulted in 60–70% pure protein. Anion exchange Chromatography through Mono-Q resulted in producing recombinant *M. tu* PPK with purity of approximately 95% and with a mass of 81 kD on SDS-PAGE (Figure 2). In the native state the protein appeared as a tetramer in gel-filtration chromatography (Text S1) similar to that reported in *E. coli* [13]. Similar quality of protein resulted from Ni-affinity column for the his-tagged protein.

### Metachromatic assay for PPK

**Figure 3:**
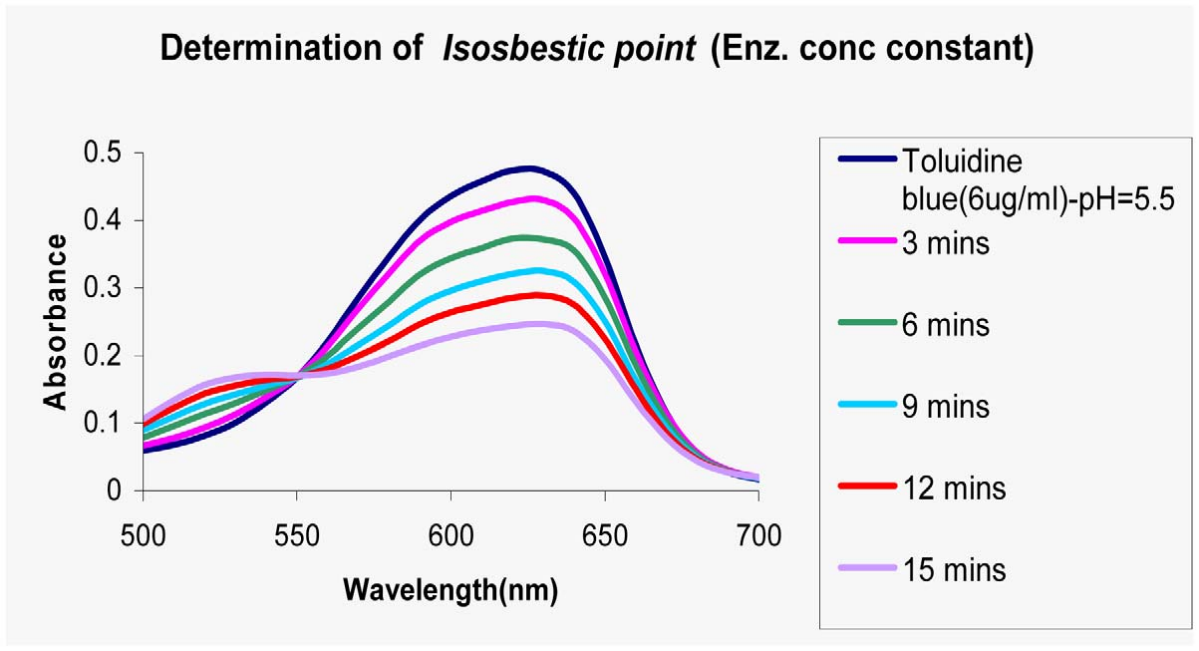
Assay for PPK activity at varying time intervals. Polyphosphate synthesis by PPK at varying time-intervals (0–15 min) was done at 37°C for 10 min and was monitored by the metachromatic assay. 1.2 ug enzyme was used for 30 ul reaction. The absorption spectra clearly shows the presence of Isosbestic Point (550 nm).

The metachromatic dye, Toluidine blue has been previously shown to be effective for the quantification of PolyP [14] and has thus been the method of choice for PPK assay. PolyP being polyanionic induces a shift in the absorption spectrum of toluidine blue from 630 to 530 nm. This shift is proportional to concentration of PolyP provided its chain-length is greater than 15 residues. The intensity of the absorption change varies not only with PolyP concentration but with the average chain-length. The latter effect can be minimized by employing PolyP of standard lengths in which case the metachromatic shift can be used in a semi-quantitative fashion [15]. To overcome these problems we adopted an alternative approach for the direct measurement of PolyP concentration. The approach involved the use of the Isosbestic Point that was observed when Toluidine blue bound to PolyP (Figure 3). The isosbestic wavelength was found to be in the range of 550–560 nm. A range, because like in many instances, isosbestic wavelength was found to vary with slight changes in pH and temperature [16]. The extinction co-efficient of Toluidine blue (unbound) was found to be 24000/M/cm [17]. Using this information, the concentration of bound dye was determined. To ascertain PolyP concentration, the knowledge of the stoichiometry of the binding of dye to PolyP was essential. This was determined indirectly by measurements of association constant Ka between the dye and polyP of various length (Table 2). The intercept of B_total_/B_free_ vs [P] plot was one and independent of the length of the PolyP, indicating 1∶1 stoichiometry between PolyP and toluidine blue.

**Table 2:** Association constant – Ka, for binding between Toluidine blue of PolyP.

Type of Poly P	Association constant Ka (M^−1^)
Type 6	0.6×10^6^
Type25	2.61×10^6^
Type 45	3.85×10^6^
Type 65	24.77×10^6^

### Optimization of PPK activity by Taguchi Protocol

As stated earlier, the assay conditions were optimised by the Taguchi method (Figure S1). The dependence of activity on divalent cation (Mg^2+^) was similar to that described for PPK of other organisms. Optimum Mg^2+^concentration was found to be 5 mM. PPK activity decreased with increasing concentrations of ammonium sulphate. However we used 10 mM Ammonium sulphate. Optimum salt concentration (NaCl) was found to be 75 mM. With increase in PolyP primer, the activity increased, but to reduce the background signal, 40 nM of PolyP-Type 6 was used. Further investigation of PPK activity revealed an optimum pH of 7.0 (Figure S2). The requirement of polyP primer was absolute for PPK activity.

### Kinetic Parameters

**Figure 4:**
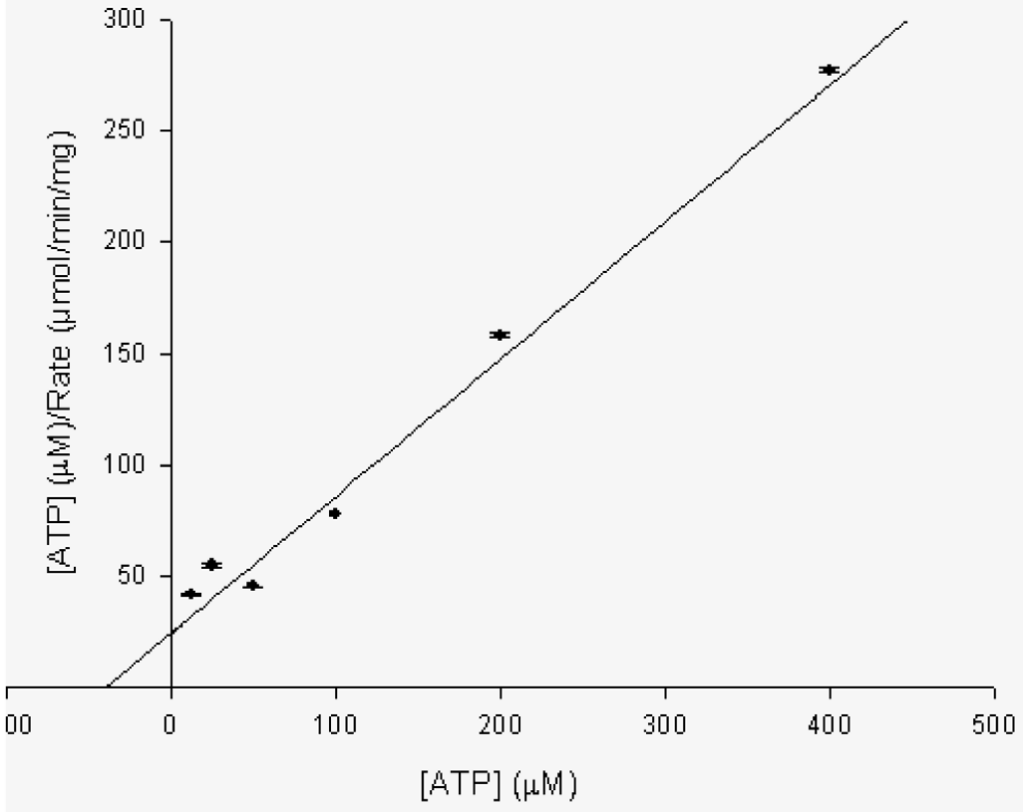
Hanes-Woolf plot for Km determination of ATP (forward assay). The Km for ATP was determined by the metachromatic assay by doing the experiment at different concentrations of ATP (12.5–400 uM) using 1.2 ug of enzyme.In this plot [S]/v is plotted against [S]. A straight line of slope 1/V max with intercepts –Km and Km/V on the abscissa and ordinate respectively is obtained.

 Kinetic analysis of PPK activity indicated a Km of approximately 40 uM as interpolated from the Hanes–Wolf plot (Figure 4). Vmax was found to be 0.56 nmole of Pi/min/mg protein. Assay with the His-tagged protein revealed similar value for Km but a lesser Vmax (20–30% less) than the untagged PPK (data not shown). The lower Vmax can be attributed to the presence of Hexa histine-tag at C-terminal end.

### HPLC assay for PPK activity

In addition to the metachromatic assay, we also used HPLC to measure ADP the other product formed in the reaction. In the reverse reaction PPK catalyzed the formation of ATP which was also monitored by HPLC. Using the stated buffer conditions we were able to resolve both ATP and ADP as two separate peaks, with retention time of 23.7 mins and 21.82 mins respectively. The Km for ATP in the forward reaction was found to be 800 uM. The large difference in the Km for ATP in the HPLC assay compared to the metachromatic method was due to the presence of product inhibition in the former which is neutralized in the latter by regenerating ATP with the aid of PEP and Pyruvate Kinase. Km for ADP in the reverse reaction was found to be approximately 100 uM (Table 3) and Vmax for the reverse reaction was found to be 0.04umolePi/min/mg protein.

**Table 3:** Kinetic constants of *M. tu* PPK.

Kinetic constant	Toluidine Blue Assay (*Forward assay*)	HPLC Assay (*Forward assay*)	HPLC Assay (*Reverse assay*)
**Km**	40 uM	800 uM	100 uM
**Kp**	-	26 uM	-

The Km for the forward reaction (formation of PolyP) was determined by both the methods (Metachromatic method and HPLC). The high Km seen in case of the HPLC method is due to ADP inhibition as the ATP-regenerating system of PEP and PK is absent in the assay. The reverse reaction was monitored only by HPLC method.

### Evaluation of Product inhibition by progress curves

**Figure 5:**
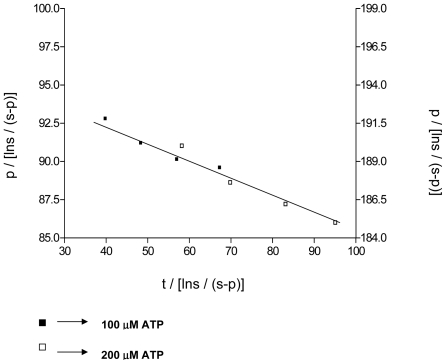
Progress curves for the determination of Kp. The data for the assay done at an ATP concentration of 100 and 200 uM, at time intervals 3, 6, 9, 12, 15 min are plotted as shown. The double ordinate plot shows that the two sets of data form a parallel line. Using the intercept in each case, Kp was calculated. Km and Vmax values were taken from the Toluidine Blue assay.

Though a powerful tool in enzyme kinetics, progress curve finds limited use in enzymology due to the problem that arises due to product inhibition. As stated earlier the difference observed in the Km of ATP in the two methods (Toluidine blue and HPLC) is due to a strong product inhibition by ADP. When the product and the substrate compete in the same space the progress curve is given by equation2. Using this kinetics the Kp of ADP was determined to be 26 uM. As postulated at various substrate values, parallel lines with similar slope and variable intercepts were obtained. Since Km, Vmax and substrate concentration was known, Kp was estimated accurately. The plot of p/[lns/(s-p)] versus t/[lns/(s-p)] indicated parallel lines for ATP concentration of 100 uM and 200 uM (Figure 5). By representing dual ordinate axis the parallel lines were shown to be overlapping.

## Discussion

PolyP has been detected in many organisms and is found to be commonly present as intracellular inclusions [18]. All the presently available data clearly indicate that PolyP plays a significant role in cellular metabolism and is really as described a “molecule for many purposes” in the cell [19].

Understanding the metabolic role of M.tu PPK would be interesting due to its several discrete functions. PPK's essential function in the stationary phase and its requirement for virulence has been shown in many organisms. The facile reversibility of the enzymatic activity of PPK may indicate that the balance between the forward and reverse reaction via a dynamic interplay between the Km of ATP and Kp of ADP might tilt the balance of the reaction one way or the other, thus providing a unique two way energy switch. This may be helpful in explaining its linkage with respect to the prolonged dormancy and its possible role in helping the bacilli while sustaining stress and nutritional deprivation. Our results indicate that a down regulation of the gene expression by antisense constructs results in bactericidal activity. However there is an important temporal factor that is incorporated. In the liquid culture down regulation of *ppk* results in stasis for about 15 days. This is followed by a rapid cidal phase. Rapid cidality associated with bacteria in late growth phase/dormant phase (beyond 14 days) correlates well with the reduction of ATP during late phase of growth. Decline in net ATP levels and a concomitant reduction of atpE mRNA encoding ATP synthase has been shown to be physiologically linked with growth phase of bacteria as well as by chemically blocking ATPase activity of mycobacteria [20]. Additionally, anaerobic *M. tuberculosis* cultures in the Wayne model at day 18 had reduced *atp*E mRNA expression and 10 fold less ATP levels compared with aerobic logarithmically growing cultures [20]. Wayne et. al. 1996 [21] have previously shown that total cellular ATP levels in dormant *M.tu* are significantly lower compared with replicating bacteria. [21]. Therefore, it appears that bacteriostatic effect of our antisense construct in actively replicating phase in the first 14 days was due to presence of higher ATP levels while sharp bactericidal activity seen at day 14 onwards following antisense downregulation of *ppk* may be related to low ATP levels during late stage of growth thus making mycobacteria more vulnerable in this phase. This was confirmed by our data on the expression levels of Mtu *ppk* in different growth phases (Table 1) which states that as compared to the constitutive gene *SigA* (Rv2703) [22], Mtu *ppk* (Rv2984) is over expressed in the log phase and there is approximately a ten fold down regulation in the stationary phase (Table 1). This is in alignment with the level of ATP during these phases. We speculate that the forward reaction is predominantly operative during the log phase and polyphosphate is generated. In contrast, the reverse reaction is operative during the stationary phase to synthesize ATP. A recent data indicates that the FoF1 ATPase inhibitor TMC 207 was inactive in the stationary phase though it had less (five fold) ATP [12]. This data apparently looks counterintuitive. However in light of our hypothesis that in stationary bacteria ATP is primarily generated through reverse PPK activity from polyphosphate and ADP and not through FoF1 ATPase, it seems logical for TMC207 to loose its potency in the stationary phase.

These results thus can be understood in light of the efficient reversibility of PPK. Compared to log phase, the vulnerability of *ppk* is even more pronounced in the stationary phase in *M. tuberculosis*. This adds another vulnerable/cidal target to the list of cidal/static mycobacterial targets recently validated by antisense down regulation [11]. It is interesting to note that in *M. smegmatis*, the rapidly growing mycobacteria *ppk* is not essential as the knockout could be generated [23]. Our results to generate knockout in *M. tuberculosis* were unsuccessful even in present of a complemented plasmid. These results are strongly indicative of the stage specific essentiality of this gene in *M. tuberculosis*. It has recently been shown that in *E. coli*, bactericidal activity of various antibiotics proceed through a common mechanism of free radical generation which leads to the upregulation of a common set of 38 genes including *ppk* [24]. It thus seems logical that down regulation of *ppk* would lead to cidality in *M. tuberculosis*. Recently a putative class II polyphosphate kinase designated as PPK 2 has been identified in mycobacteria [25]. It was seen that PPK2 catalyzes the synthesis of GTP from GDP using polyP rather than ATP as phosphate donor. This data indicated that PPK2 could not biochemically complement PPK1 [25].

A pre-requisite for PPK to be a validated drug target in *M. tuberculosis*, is the elucidation of its role in survival of the bacteria *in-vivo*, especially during the latent phase, and finally in the human host. The fact that this gene is absent in human brings in a unique selectivity. The biochemical pathways in *M. tuberculosi*s that are involved in latency are still elusive. But it seems logical that PPK due to its facile reversibility and its stage specific essentiality would turn out to be a prime candidate. Our understanding is that PPK in the log phase does the non essential role of synthesizing polyphosphate utilizing the excess of ATP. In the non replicating phase (stationary, dormant or latent) it does the essential role of producing ATP from the stored polyphosphate. Our detailed kinetic analysis of this reaction supports this hypothesis. Finally we have also developed the Toluidine blue assay for monitoring the PolyP concentration by bringing in the observed phenomenon of Isosbestic point. This assay has been suitably converted to a high throughput microtitre plate format that is suitable for screening potential inhibitors. The selectivity of these inhibitors would be in contrast to TMC207, PPK inhibitors would be cidal in the stationary phase. Molecules that induce cidality in non replicating mycobacteria would be a key component in combination antitubercular therapy.

## Materials and Methods

### Antisense *ppk* (ppk-AS) in *M. tuberculosis*

To test the vulnerability (survivability under down regulation) of *ppk*, the full length gene (Rv2984 from *M. tu*. annotated as *ppk* in KEGG) was cloned in antisense orientation in the IPTG inducible mycobacterial shuttle vector pAZI9018b [11] in the *Bam*HI and *Nde*I site. The *ppk* antisense clone was transformed into *M. tuberculosis* by electroporation and equal amount of transformants were plated at various concentration of IPTG (ranging 0, 10, 100 and 1000 uM). Transformants growing in uninduced plates were grown to about 10^7^ cfu/ml and antisense *ppk* expression was induced by IPTG (ranging from 0 to 1000 uM). Survivability of the bacilli during a five week period (∼35 generations) was monitored in triplicate by plating for colony forming units (cfu). Details of the transformation and survival kinetics are discussed earlier [11].

### Regulation of *M. tuberculosis ppk* gene expression during different phases of growth

An *M. tuberculosis* culture grown in 7H9 complete medium (with 0.05% Tween and ADC supplement) was harvested in log phase (5 days), late log/early stationary phase (28 days) and late stationary phase (63 days). One ml of Trizol® was added to the cell pellets (from 5 ml culture) to stabilize and arrest the mRNA. Cells were disrupted by bead beating using 0.1 mm diameter zirconium beads (Biospec), followed by a 5 min centrifugation at 14,000 g. Total RNA was isolated and prepared for transcriptome analysis by qRTPCR as reported earlier [11]. Expression level of Mtu *ppk* (Rv2984) was estimated in comparison to the house-keeping gene Mtu *sigA* (Rv2703) in different phases of growth [22]. RTPCR was performed in quadruplicate using SYBR Green chemistry and data was analyzed as reported [11]. The primers used for this assay were: sigART(F) 5′-TCGAGGTGATCAACAAGCTG-3′, sigART(R) 5′-CTGCAGCAAAGTGAAGGACA-3′, ppkRT(F) 5′-. TTACAACAGC AAGACAGCACG-3′, and ppkRT (R) 5′-CATCAACAAGGGCATTCATCT-3′.

### Construction of recombinant PPK clones

The gene Rv2984 from *M. tu*. was amplified using the primers ppk-F (5′atgagcaatgatcgcaaggtg 3′) and ppk-H (5′cccaagcttggggctgcggtgccgttccatc 3′). The amplified product was digested with *Hind*III and was cloned into *Nco*I blunt-*Hind*III site of pET-21d (Amp^r^, C-terminal hexa His-tag) to form pAZ-2 clone. The clones were confirmed by DNA sequencing. For the cloning of untagged *ppk*, the gene cloned in pET-21D was amplified with a stop codon and recloned cloned in the same vector to form pAZ-1.

### Reagents

Pyruvate kinase, ATP, PolyP (Type 6, 25, 45, 65, 75), Toluidine blue and IPTG were procured from Sigma Chemicals, restriction enzymes from New England Biolabs and Bangalore Genei (India). PCR was done using Expand polymerase from Roche.

### Strains

Strains used were *E. coli* MOS blue, *E. coli* BL-21(DE3) and *M. tuberculosis* H37Rv ATCC 27294.

### Purification of recombinant *M. tu* PPK

*E. coli* BL-21 (DE3) cells transformed with pAZ (1 or 2) clones were grown in 500 ml LB (100 ug/ml ampicillin) at 37°C till 0.6 O.D_600_ after which the cells were downshifted and grown at 20°C O/N for 16 hrs. to achieve soluble overexpression of PPK. Induction by IPTG was not required. Harvested cells were suspended in 1/10^th^ culture volume of Buffer A (−50 mM Tris, pH-7.5, 1 mM PMSF, 1 mM EDTA, 1 mM DTT, 10% glycerol) and lysed by sonication till O.D_600_ reached 1/10^th^ the initial O.D. The homogenate was spun at 50,000× g for 30 mins to remove cell-debris.

The lysate was then subjected to ammonium sulphate precipitation (35% v/v) by addition of appropriate volume of saturated ammonium sulphate solution. After 15 mins, the suspension was spun at 30,000× g for 20 min. The pellet was stored in 20°C, if not processed immediately.

### Purification of untagged PPK

The ammonium sulphate pellet was suspended in appropriate volume of Buffer A and loaded onto 8 ml Mono-Q column (Amersham Life Sciences) equilibrated with 10 column volumes (80 ml) of Buffer A. Following a wash with 3 column volumes of buffer A, the protein of interest was eluted with 10 column volumes of Buffer B (Buffer A+1M NaCl-Linear gradient) and the peak fractions were pooled. Protein yields were estimated by Bradford's method and purity was checked by SDS-PAGE. The purified PPK was then aliquoted and stored in −70°C.

### Purification of His-tagged PPK

The ammonium sulphate pellet was suspended in an appropriate volume of Buffer C (50 mM NaH_2_PO_4_,10 mM imidazole - pH = 8.0) to get a protein concentration of 1–2 mg/ml. The suspension was spun at (48,500× g) and NaCl was added till conductance was identical to Buffer D (50 mM NaH_2_PO_4_, 20 mM imidazole, 300 mM NaCl, pH = 8.0). It was then loaded onto Ni–NTA Hi-trap (Qiagen) column that had been pre-equilibrated with 10 column volumes of Buffer D. The column was washed with 3 column volumes of buffer D and eluted with Buffer E (50 mM NaH_2_PO_4_, 300 mM NaCl, 300 mM imidazole, pH = 8.0). The eluted fraction was dialysed overnight against Buffer F (50 mM Tris pH = 7.5,10 mM KPO_4_ - pH-7.0, 4 mM MgCl_2_, 1 mM PMSF, 20% glycerol). Protein yields were estimated by Bradford's method and purity was checked by SDS-PAGE. The purified PPK was then aliquoted and stored in −70°C.

### Taguchi optimization for PPK activity

In order to standardize the activity of *M.tu* PPK, it is essential to identify the condition under which the activity is optimum. The standard parameters that one optimizes for activity are the PolyP, concentration of Mg^2+^, NaCl and NH_4_SO_4_. Instead of the standard two variable checkerboard strategies, we have used the multiparametric Taguchi methods for optimization [26]. Though this powerful statistical method has been successfully used for optimization of PCR conditions [27], it does not find any other mention in classical enzymology. To determine the optimum conditions for the four parameters we need to do nine individual experiments. The conditions for each variable chosen is a ‘guesstimate’ so that the optimum condition would probably lie between the maximum and the minimum value of the variable. The velocity of the enzyme reaction in each experiment was used to estimate the effect of each component on the enzyme reaction. This was done by using a quadratic loss function given by the equation
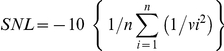



Where vi is the velocity of the reaction in the *ith* experiment. SNL is plotted against each component, and the optimum reaction conditions were chosen by identifying the value of the component which maximized SNL according to the polynomial regression curve, y = mx + nx^2^ (Figure S1).

### Assay for PPK activity

PPK activity (ATP-driven PolyP synthesis) was determined at 37°C by detecting PolyP formation. The assay mixture (30 ul) consisted of 50 mM Tris-HCl pH-7.5, 10 mM NH_4_SO_4_,10 mM KPO_4_,pH-7.0,4 mM MgCl_2_, 50 mM NaCl, 150 uM ATP and 40 nM Type 6 PolyP (Sigma). Each of the reactions (30 ul) were terminated by adding to 270 ul of Toluidine blue and the absorption ratio (530/630) nm was calculated in each case. To overcome product inhibition by ADP formed during the PPK reaction, 6 mM PEP and 10 U of pyruvate kinase was included in the assay mix to bring about ATP regeneration [14], [18], [28]. The reaction was initiated by the addition of the enzyme (0.5 ug) and incubated at 37°C for 10 min. The increase in polyP concentration was then determined by the method outlined below. The kinetic parameters, Km and Vmax were determined by conducting the assay in triplicate at 6 different ATP concentrations. The enzyme preparations were additionally checked by Luciferin–Luciferase assay (Text S2).

### Determination of PolyP formed during assay of PPK by metachromasy

PPK activity was monitored by the metachromatic shift. This method is based on the shift in the absorbance maxima of the basic dye Toluidine blue from 630 nm to 530 nm when PolyP or any polyanion binds to the dye. As the polyanionic charge increases, there is a concomitant increase in the magnitude of the metachromatic shift [14], [28], [29]. For quantifying the amount of PolyP formed, the entire assay mix (30 ul) was added to 270 ul of dye solution (6 ug/mL Toluidine blue in 50 mM acetate buffer pH-5.5). The metachomatic shift was monitored by a wavelength scan from 500 to 700 nm. The resultant absorption spectra revealed the presence of an Isosbestic Point - a wavelength at which two or more components have the same extinction co-efficient. The absorbance at isosbestic point can be used to determine the total concentration of the two components which could predictably be the two forms of toluidine blue – the PolyP-bound Toluidine blue – [b] and the unbound Toluidine blue - [u]. The concentration of each of forms was determined using the generic formula [30].
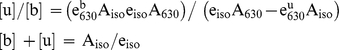



Thus, knowing the concentration of bound dye molecule, the concentration of PolyP can be determined by dividing the former with the stoichiometric factor (binding ratio).

### Determination of binding constant of Toluidine blue to PolyP

To evaluate the binding constant (Ka) between polyP of various length (P6, P25, P45 and P65) and Toluidine blue, concentration of PolyP was varied while the dye was kept constant. The association constant Ka  =  [PB]/[P] [B], where PB is the concentration of polyphosphate bound dye while P and B free polyphosphate and dye concentrations. By simple transposition we have the equation: {[PB] + [B]}/[B]  = 1+ Ka [P].

Thus in an experiment where the concentration of Toluidine blue is kept fixed and the polyphosphate concentration varied if we plot B_total_/B_free_ vs [P] we would get a straight line where the slope would give Ka. The intercept of one is a diagnostic of the binding which would indicate a one to one stoichiometry between PolyP and the dye.

### Direct measurement of ADP formation by PPK through HPLC-Reverse-Phase Chromatography

The substrate ATP and the product ADP were separated on a C-18-silica column using a binary gradient of Methanol and the ion pairing agent TBAHS (10 mM, pH = 6) as the mobile phase, 50 ul volume of standard PPK reaction was injected and separation monitored at 260 nm at a flow-rate of 1 ml/min. Run-time was 30 minutes. Prior to HPLC analysis, different concentrations of ADP were run and a standard curve was plotted – Area under the peak against amount of ADP. Using this curve, the amount of ADP in the assay samples was determined. The conditions for the assay was the same except for the omission of the ATP-regenerating system (PEP and Pyruvate kinase).

### Evaluating product inhibition by Progress Curve

Many enzymes are inhibited by their product and hence initial velocity measurement is the method of choice for evaluating the kinetic constants [31]. Since progress curve goes into the relatively late phase of the reaction where substantial product is already formed the kinetics gets modified and simplified analysis by progress curve may become erroneous. However when the product inhibition is significant and suitable continuous kinetic assay are unavailable, initial velocity measurements that are devoid of product inhibition are difficult to perform. We can then use the modified form of progress curve that takes product inhibition into account.

Now, v/V_m_ =  S/(S+Km) is the standard form of the MM equation. We have, v =  dp/dt where p is the product; also the substrate conc. at time t is S and the initial conc is s

hence S =  s-p.

Using these changes the differential form of the MM equation is given by 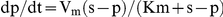 or, 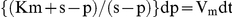.

In presence of product inhibition, since the product competes with the substrate we can substitute Km by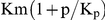 or, 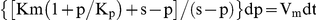 or, 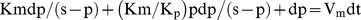.

Integrating we have: 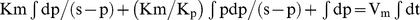 or, 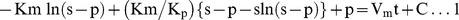 at t = 0 p = 0 thus using these boundary conditions we have, substituting the value of C in eqtn1 we have: 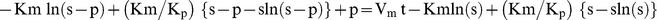 or, 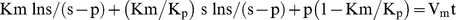 or, 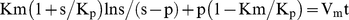.

Transposing 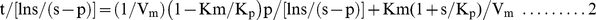. Thus plotting 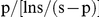 vs 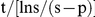 will give a straight line with a slope of 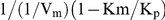 and intercept 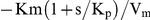. This indicates that at various values of s we will have parallel lines variable intercepts.

Since the values of Km, V_m_ and s are known, the value of K_p_ can be accurately estimated.

An experiment was designed wherein assay was done at different substrate concentrations for different time intervals. The assay product was analyzed by HPLC. From this data, p and s-p values were calculated and required graphs plotted.

### Assay for reverse PPK activity

PolyP-driven ATP synthesis of PPK was detected at 37°C by monitoring the formation of ATP using HPLC. The conditions used were same as that used for the forward reaction (PolyP synthesis). The assay-mix consisted of 50 mM Tris-HCl pH-7.5, 10 mM NH_4_SO_4_, 10 mM KPO_4_, pH-7.0, 4 mM MgCl_2_, 50 mM NaCl, 400 uM ADP and 5.5 uM Type 75 PolyP (Sigma). The assay was done for 5 mins at 37°C with the reaction being stopped by the addition of 5 mM EDTA and 50 ul of the assay-mix was injected onto the column.

Amount of the product formed was calculated from an ATP standard curve and Km for ADP was determined by monitoring the assay at different ADP concentrations.

### Determination of oligomeric structure of PPK

Size-exclusion chromatography was used to measure the oligomeric nature of PPK. About 200 ug of purified PPK was loaded onto Superose-6 (HR-Amersham Pharmacia) equilibrated with Buffer G (50 mM Tris-pH = 7.5, 150 mM NaCl, 4 mM MgCl_2_ and 10% glycerol) along with protein MW standards (BioRad).

## Supporting Information

Text S1 Determination of oligomeric structure of PPK: Size-exclusion chromatography was used to measure the oligomeric nature of PPK. About 200 µg of purified PPK was loaded onto Superose-6 (HR-Amersham Pharmacia) equilibrated with Buffer G (50 mM Tris-pH = 7.5, 150 mM NaCl, 4 mM MgCl2 and 10% glycerol) along with protein MW standards (BioRad).
(0.86 MB TIF)

Text S2 Assay for PPK activity by Luciferin-Luciferase method
(0.77 MB TIF)

Figure S1 PPK activity. Effects of reaction components on the activity of PPK as determined by polynomial regression. The signal here represents the 530/630 ratio. An average of the ratios was taken for each reaction component at each level of concentration tested. The assay was done based on an orthogonal array (total of 9 different reactions).
(0.63 MB TIF)

Figure S2 Determination of Optimum pH for Mtu PPK. Using the metachromatic assay, the optimum pH for PPK activity was determined by doing the assay with 50 mM Tris. The 530/630 ratio is plotted against pH. The assay was done with 1.2 µg of purified PPK at 37°C for 10 min.
(0.56 MB TIF)
