# Supplementary figures and images for: Polyphosphate Kinase from M. tuberculosis: An Interconnect between the Genetic and Biochemical Role
Source: PLoS One. 2010 Dec 15;5(12):e14336. doi: 10.1371/journal.pone.0014336 (PMC3002279; doi:10.1371/journal.pone.0014336)

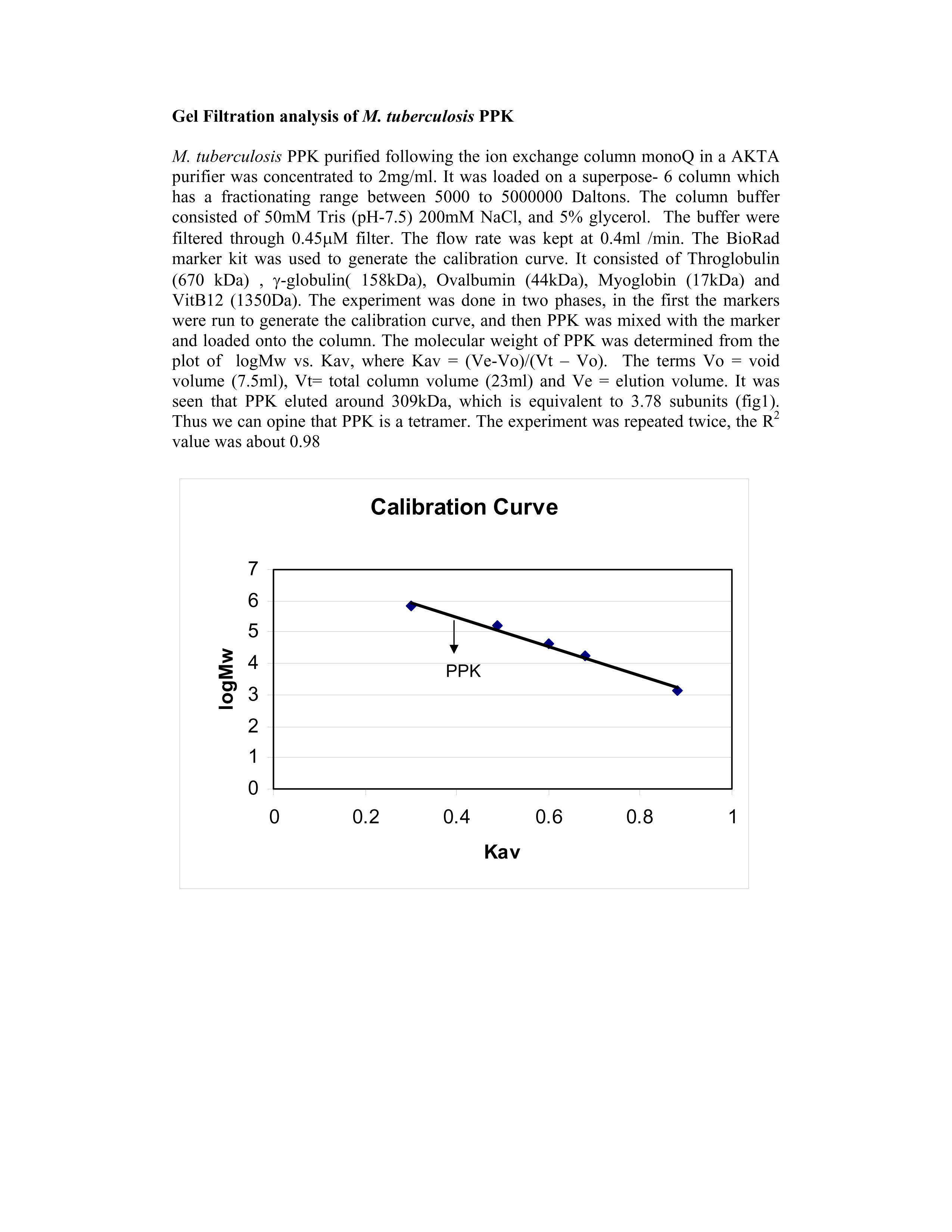

Supplement: Text S1 — Determination of oligomeric structure of PPK: Size-exclusion chromatography was used to measure the oligomeric nature of PPK. About 200 µg of purified PPK was loaded onto Superose-6 (HR-Amersham Pharmacia) equilibrated with Buffer G (50 mM Tris-pH = 7.5, 150 mM NaCl, 4 mM MgCl2 and 10% glycerol) along with protein MW standards (BioRad). (0.86 MB TIF) [file pone.0014336.s001.tif]

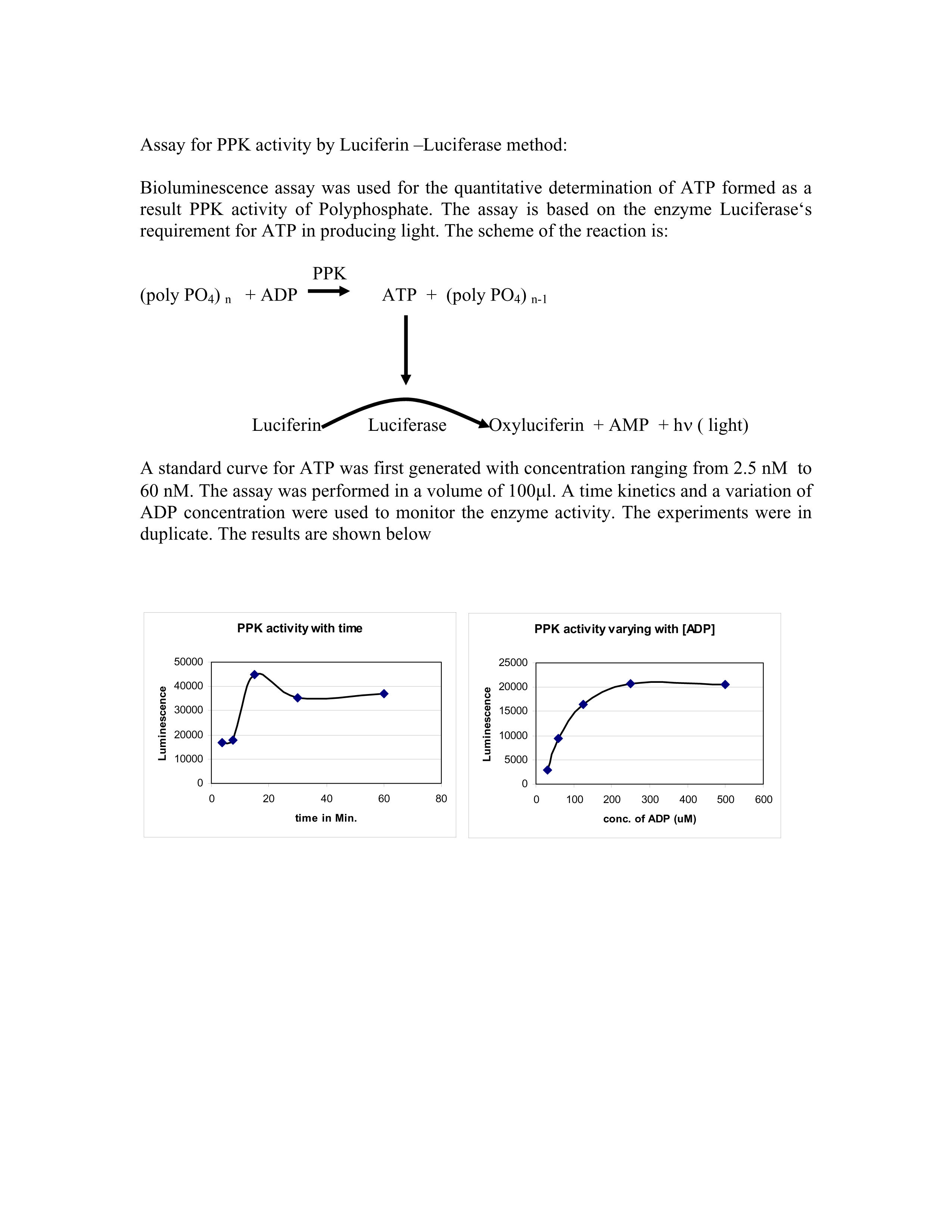

Supplement: Text S2 — Assay for PPK activity by Luciferin-Luciferase method (0.77 MB TIF) [file pone.0014336.s002.tif]

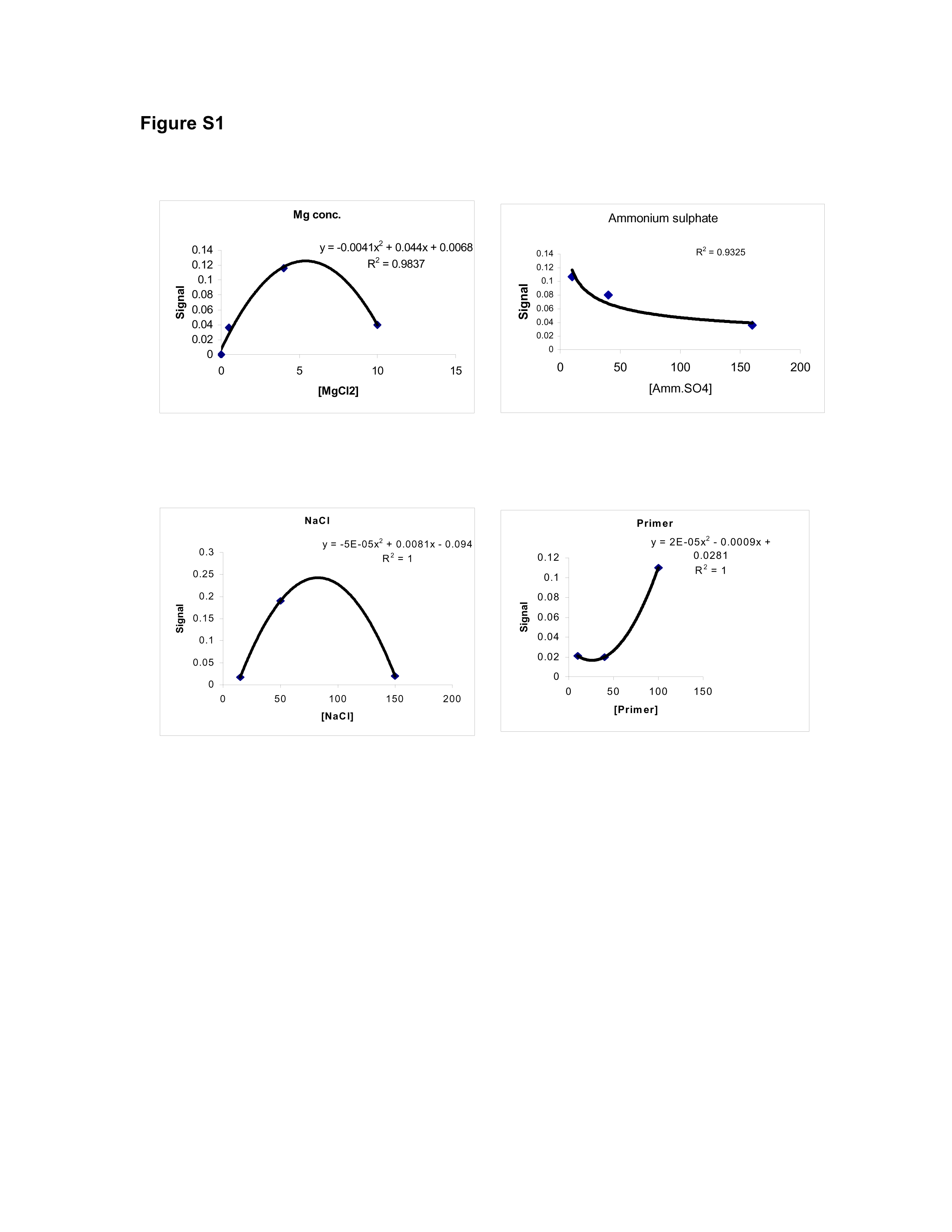

Supplement: Figure S1 — PPK activity. Effects of reaction components on the activity of PPK as determined by polynomial regression. The signal here represents the 530/630 ratio. An average of the ratios was taken for each reaction component at each level of concentration tested. The assay was done based on an orthogonal array (total of 9 different reactions). (0.63 MB TIF) [file pone.0014336.s003.tif]

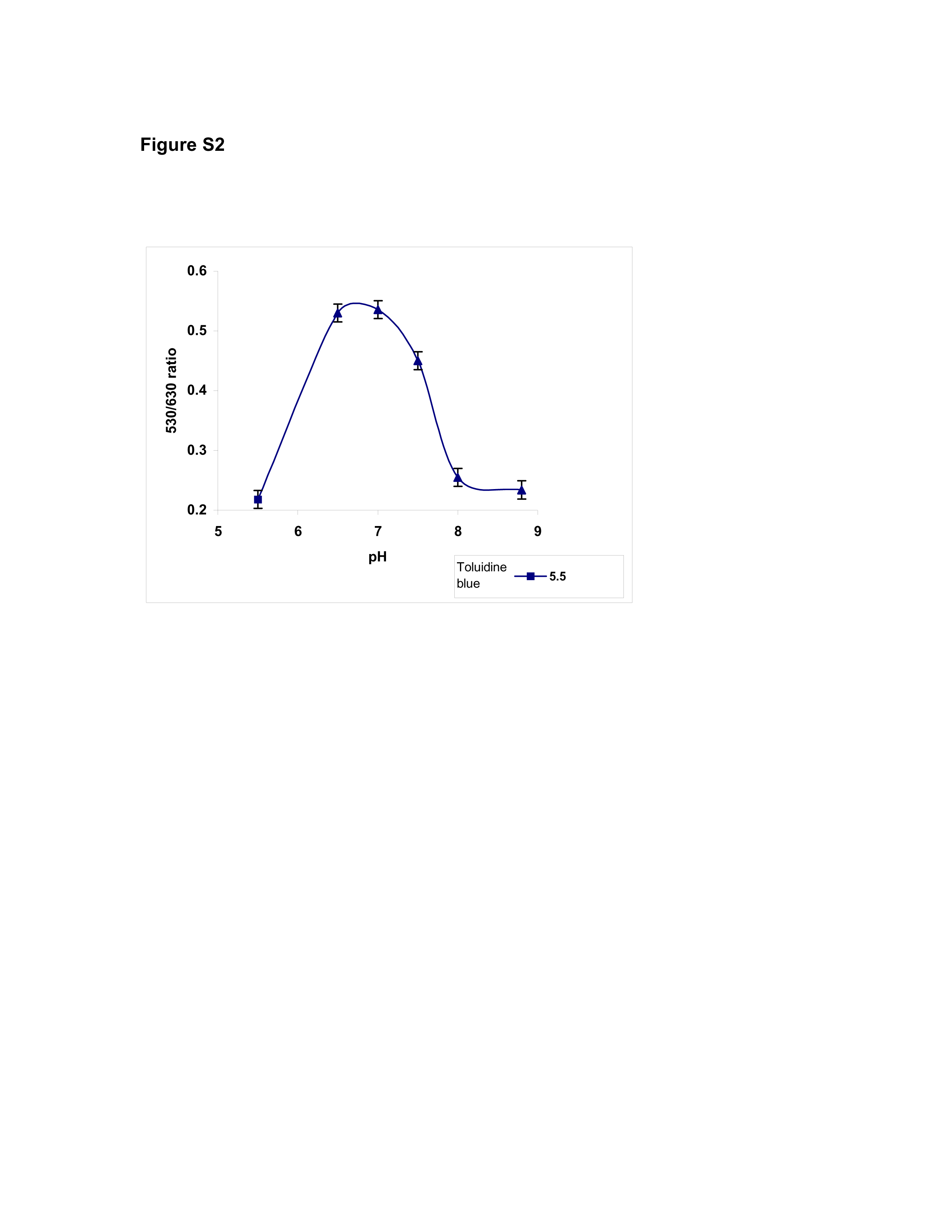

Supplement: Figure S2 — Determination of Optimum pH for Mtu PPK. Using the metachromatic assay, the optimum pH for PPK activity was determined by doing the assay with 50 mM Tris. The 530/630 ratio is plotted against pH. The assay was done with 1.2 µg of purified PPK at 37°C for 10 min. (0.56 MB TIF) [file pone.0014336.s004.tif]
